# Salt Substitutes—An Important Tool to Increase Potassium and Reduce Sodium Intakes?

**DOI:** 10.3390/nu15122647

**Published:** 2023-06-06

**Authors:** Rachael Mira McLean

**Affiliations:** Department of Preventive & Social Medicine, Dunedin School of Medicine, University of Otago, P.O. Box 56, Dunedin 9016, New Zealand; rachael.mclean@otago.ac.nz; Tel.: +64-3479-9581

Potassium is an essential mineral and is the main cation in intracellular fluid. The World Health Organization (WHO) recommends that adults consume at least 90 mmol/day (3510 mg/day) of potassium from foods [1]. The relationship between sodium and potassium at the cellular level is responsible for many essential functions, including maintaining fluid balance. This Special Issue of *Nutrients*, entitled ‘The Role of Dietary Potassium in Human Health’, highlights the important relationship between potassium, sodium, and health, and provides valuable information, particularly related to blood pressure. This informs a key strategy for reducing dietary sodium and increasing dietary potassium to improve health—the use of potassium-enriched, reduced-sodium salt as a substitute for ordinary table salt (sodium chloride).

In 2013, The World Health Organization (WHO) set a global target of reducing population salt (sodium) intake by 30% by 2025 to mitigate health issues such as high blood pressure and cardiovascular disease [2]. The WHO also recommends a sodium–potassium molar ratio of <1 to help lower blood pressure [3]. Progress towards these targets has been slow, with ongoing high sodium and low potassium intakes worldwide [4,5]. A range of strategies have been proposed to reduce sodium intakes, including the reformulation of processed foods, improved food labelling and consumer education [6]. Recent research has focused on the use of reduced-sodium salt substitutes (commonly known as low-sodium salt substitutes) to reduce sodium intake worldwide. This may be particularly effective in countries and communities where discretionary salt (salt used in cooking and at the table) constitutes a high proportion of total salt intake. Many of these products replace sodium chloride with potassium chloride, thereby having the added benefit of increasing potassium intake and improving the sodium–potassium ratio. Cluster randomized trials conducted in China (where discretionary salt constitutes between 40% and 75% of total salt intake [7]) have recently been conducted for the replacement of table salt with potassium-enriched, reduced-sodium salt substitutes in community and residential care facilities. These trials demonstrate an association between the use of these salt substitutes and reductions in systolic blood pressure and cardiovascular events [8,9]. A recent systematic review using a Cochrane meta-analysis of 26 randomised controlled trials, which involved the use of reduced-sodium salt substitutes, demonstrated a reduction in systolic blood pressure of 4.76 mmHg (95% confidence interval (CI) 3.50, 6.01). This level of blood pressure reduction would be considerably beneficial if the interventions were applied at a population level [10].

In this Special Issue, Ajenikoko et al. provide a comprehensive review of evidence to support potential policies and strategies in order to increase the global intake of potassium-enriched, reduced-sodium salt [11]. They identify a number of barriers in the current environment, including the lack of awareness, availability, and affordability of these products, as well as concerns regarding potential taste effects (some studies show that, if the proportion of potassium chloride exceeds 30%, consumers may experience a ‘metallic taste’). Furthermore, if global efforts to maintain sufficient iodine via salt iodisation are to be successful, salt substitutes should also be iodised. Securing key stakeholders, including governments, communities, civil society, and the food industry, is key to maximizing the potential of these products.

Concerns have been expressed about the potential of potassium-enriched salt substitutes to increase serum potassium in vulnerable individuals (people with kidney failure, and those on potassium-sparing diuretics), which carries a risk of fatal cardiac arrythmias [12]. Although, at a population level, blood pressure and cardiovascular benefits may outweigh the risks associated with hyperkalaemia [13], some researchers have suggested that warning labels should be used on these products, and dietary advice for people known to have impaired kidney function should include information on these risks [11]. In this Special Issue, a modelling study on the potential harm of using potassium-enriched, reduced-sodium salt in bread at various concentrations in Australia suggests that, for those with chronic kidney disease, the maximum recommended potassium intake levels would be exceeded [14].

The extent to which potassium supplementation may benefit or harm those with mild to moderate kidney disease has also been questioned. While there may be theoretical benefits of a high potassium intake in those with mild kidney disease (via reduced blood pressure maintaining remaining kidney function), Turban et al. [15] provide valuable evidence that this is unlikely to be the case in practice. Their randomised, controlled cross-over feeding trial of a low vs high potassium diet in adults with mild to moderate (stage 3) chronic kidney disease showed that the higher potassium diet was not associated with blood pressure lowering. The higher potassium diet was associated with increased serum potassium, with two participants on the higher potassium diet developing hyperkalaemia.

Although much of the current research has focused on the use of salt substitutes as replacements for discretionary salt, many populations (particularly those in Western countries) consume only a small proportion of total salt intake as discretionary salt [7]. The use of reduced-sodium salt substitutes in processed foods (such as bread) and sauces needs to be further explored, if these substitutes are to be successfully used in this context. Umeki et al. [16] provide new information on the potential use of this strategy in a Japanese population. These authors conducted a randomised controlled trial of men aged 35 and over with moderately elevated blood pressure, but who were not taking antihypertensive medications, who were provided with packed lunches 5 days a week for 6 weeks. The study involved the preparation of meals using potassium-enriched, reduced-sodium salts and seasonings, including reduced-sodium instant miso soup. At 6 weeks, the study found a greater reduction in systolic blood pressure in the intervention group (mean difference: −2.1 (95% CI −3.6, 0.6)) mmHg. Unfortunately, the participants were not asked directly about the palatability of the food products; however, the authors state that consumption of the food products was the same in both groups, suggesting that palatability was not an issue.

The most important sources of dietary potassium from foods include fruits, vegetables, legumes and wholegrains [17]. Kumssa et al. [17] estimated per capita supplies at a national level using food balance sheets from the Food and Agriculture Organization of the United Nations written between 1961 and 2017. Food balance sheets aim to provide “a comprehensive picture of the pattern of a country’s food supply for a specified reference period” [18]. While the use of food balance sheets does not provide accurate information on an individual level, the study shows that, for most countries, potassium supplies in food were adequate compared to local nutrient reference values, with supplies substantially increasing across the period in countries in East Asia.

Potassium intake at recommended levels from foods remains an important strategy for maintaining health, particularly with regard to lowering blood pressure and the risk of cardiovascular disease [1]. Choosing foods that are high in potassium is associated with improvements in overall diet quality, as they are often high in fiber and other nutrients. Potassium-enriched, reduced-sodium salt substitutes are a valuable additional tool to reduce sodium and increase potassium intake, particularly when used to replace table salt (sodium chloride) in discretionary settings.

## References

[1] World Health Organization (2012). Guideline: Potassium Intake for Adults and Children.

[2] World Health Organization (2013). Global Action Plan for the Prevention and Control of Noncommunicable Diseases 2013–2020.

[3] World Health Organization (2012). Guideline: Sodium Intake for Adults and Children.

[4] Santos J.A., Tekle D., Rosewarne E., Flexner N., Cobb L., Al-Jawaldeh A., Kim W.J., Breda J., Whiting S., Campbell N. (2021). A Systematic Review of Salt Reduction Initiatives Around the World: A Midterm Evaluation of Progress towards the 2025 Global Non-Communicable Diseases Salt Reduction Target. Adv. Nutr..

[5] Ginos B.N., Engberink R.H.O. (2020). Estimation of Sodium and Potassium Intake: Current Limitations and Future Perspectives. Nutrients.

[6] World Health Organization (2016). The SHAKE Technical Package for Salt Reduction.

[7] Bhat S., Marklund M., Henry M.E., Appel L.J., Croft K.D., Neal B., Wu J.H. (2020). A Systematic Review of the Sources of Dietary Salt Around the World. Adv. Nutr..

[8] Neal B., Wu Y., Feng X., Zhang R., Zhang Y., Shi J., Zhang J., Tian M., Huang L., Li Z. (2021). Effect of Salt Substitution on Cardiovascular Events and Death. N. Engl. J. Med..

[9] Yuan Y., Jin A., Neal B., Feng X., Qiao Q., Wang H., Zhang R., Li J., Duan P., Cao L.E. (2023). Salt substitution and salt-supply restriction for lowering blood pressure in elderly care facilities: A cluster-randomized trial. Nat. Med..

[10] Bibbins-Domingo K., Chertow G.M., Coxson P.G., Moran A., Lightwood J.M., Pletcher M.J., Goldman L. (2010). Projected effect of dietary salt reductions on future cardiovascular disease. N. Engl. J. Med..

[11] Ajenikoko A., Ide N., Shivashankar R., Ge Z., Marklund M., Anderson C., Atun A., Thomson A., Henry M.E., Cobb L.K. (2021). Core Strategies to Increase the Uptake and Use of Potassium-Enriched Low-Sodium Salt. Nutrients.

[12] Marklund M., Singh G., Greer R., Cudhea F., Matsushita K., Micha R., Brady T., Zhao D., Huang L., Tian M. (2020). Estimated population wide benefits and risks in China of lowering sodium through potassium enriched salt substitution: Modelling study. BMJ.

[13] Scientific Advisory Committee on Nutrition, Committee on Toxicity (2017). Potassium-Based Sodium Replacers: Assessment of the Health Benefits and Risks of Using Potassium-Based Sodium Replacers in Foods in the UK. https://www.gov.uk/government/publications/sacn-cot-statements-on-potassium-based-sodium-replacers.

[14] Morrison R., Stanford J., Lambert K. (2021). Dietary Modelling to Explore the Impact of Potassium Chloride Replacement for Sodium in Bread for Adults with Chronic Kidney Disease. Nutrients.

[15] Turban S., Juraschek S.P., Miller III E.R., Anderson C.A., White K., Charleston J., Appel L.J. (2021). Randomized Trial on the Effects of Dietary Potassium on Blood Pressure and Serum Potassium Levels in Adults with Chronic Kidney Disease. Nutrients.

[16] Umeki Y., Hayabuchi H., Adachi H., Ohta M. (2021). Feasibility of Low-Sodium, High-Potassium Processed Foods and Their Effect on Blood Pressure in Free-Living Japanese Men: A Randomized, Double-Blind Controlled Trial. Nutrients.

[17] Kumssa D.B., Joy E.J.M., Broadley M.R. (2021). Global Trends (1961–2017) in Human Dietary Potassium Supplies. Nutrients.

[18] Food and Agriculture Organization of the United Nations (2001). Food Balance Sheets: A Handbook.

